# Urinary I-FABP, L-FABP, TFF-3, and SAA Can Diagnose and Predict the Disease Course in Necrotizing Enterocolitis at the Early Stage of Disease

**DOI:** 10.1155/2020/3074313

**Published:** 2020-03-03

**Authors:** Stepan Coufal, Alena Kokesova, Helena Tlaskalova-Hogenova, Barbora Frybova, Jiri Snajdauf, Michal Rygl, Miloslav Kverka

**Affiliations:** ^1^Institute of Microbiology of the Czech Academy of Sciences, v.v.i., Prague 142 20, Czech Republic; ^2^Department of Pediatric Surgery, 2nd Faculty of Medicine, Charles University in Prague and Motol University Hospital, Prague 150 06, Czech Republic

## Abstract

Necrotizing enterocolitis (NEC) is a severe gastrointestinal disease affecting mainly preterm newborns. It is characterized by unexpected onset and rapid progression with specific diagnostic signs as *pneumatosis intestinalis* or gas in the portal vein appearing later in the course of the disease. Therefore, we analyzed diagnostic and prognostic potential of the markers of early NEC pathogenesis, such as excessive inflammatory response (serum amyloid A (SAA)) and gut epithelium damage (intestinal and liver fatty acid-binding protein (I-FABP and L-FABP, respectively) and trefoil factor-3 (TFF-3)). We used ELISA to analyze these biomarkers in the urine of patients with suspected NEC, either spontaneous or surgery-related, or in infants without gut surgery (controls). Next, we compared their levels with the type of the disease (NEC or sepsis) and its severity. Already at the time of NEC suspicion, infants who developed NEC had significantly higher levels of all tested biomarkers than controls and higher levels of I-FABP and L-FABP than those who will later develop sepsis. Infants who will develop surgery-related NEC had higher levels of I-FABP and L-FABP than those who will develop sepsis already during the first 6 hours after the abdominal surgery. I-FABP was able to discriminate between infants who will develop NEC or sepsis and the SAA was able to discriminate between medical and surgical NEC. Moreover, the combination of TFF-3 with I-FABP and SAA could predict *pneumatosis intestinalis*, and the combination of I-FABP, L-FABP, and SAA could predict gas in the portal vein or long-term hospitalization and low SAA predicts early full enteral feeding. Thus, these biomarkers may be useful not only in the early, noninvasive diagnostics but also in the subsequent NEC management.

## 1. Introduction

Necrotizing enterocolitis (NEC) is a severe acute gastrointestinal disease affecting mainly preterm newborns. The pathophysiology of NEC remains poorly understood. The main risk factors of NEC are immaturity of gut barrier and immune system together with enteral feeding and abnormal microbial colonization of the gut. On the other hand, breastfeeding represents an important factor protecting from NEC [1, 2]. Thanks to its unique composition, mother's milk not only accelerates gut barrier maturation but also protects neonatal gut from infection. The former is achieved mainly by numerous growth factors and the later by antimicrobial factors (e.g., lactoferrin and lysozyme) and by secretory IgA, which could bind microbes in antigen-specific and nonspecific manner by Fab or glycans, respectively [3, 4].

With overall incidence of 1 to 3 cases per 1000 live births and mortality as high as 50% [5, 6], NEC is one of the leading causes of morbidity and mortality in neonatal intensive care units [7]. The early recognition and proper treatment can, however, improve the clinical outcomes [8]. The current diagnosis of NEC is based on the combination of clinical, laboratory, and radiologic or sonographic findings, which are defined by modified Bell's staging criteria [9]. But NEC has often rapid onset and progression with nonspecific early signs, which may delay the NEC treatment by misdiagnosing it as neonatal sepsis or other medical emergency. While there are specific signs for NEC, such as *pneumatosis intestinalis* or gas in the portal vein, they appear rather later in the disease course and their absence must be interpreted with caution [10]. Therefore, the identification of specific biomarkers for early diagnosis of NEC is strongly needed.

The best biomarkers should be specific for the early steps in NEC pathogenesis. Since several studies demonstrated the advantages of biomarker combination in the NEC diagnosis, we combined analysis of several noninvasive markers of excessive inflammatory response and destruction of the gut mucosa, all typical features of NEC pathogenesis [11].

Previously, we showed that urinary intestinal fatty acid-binding protein (I-FABP) can distinguish NEC from sepsis in early stage of the disease [12]. Fatty acid-binding proteins (FABPs) are small (14-15 kDa) tissue-specific cytoplasmic proteins involved in the metabolisms of fatty acids. The I-FABP constitutes up to 2% of cytoplasmic protein content in the mature enterocyte. The liver fatty acid-binding protein (L-FABP) is expressed in similar pattern of tissue distribution along the duodenal-colonal axis, but with the higher tissue content than I-FABP (up to 40-fold). During the enterocyte death, both I-FABP and L-FABP are released into the circulation and thus can be used as a marker of intestinal damage [13, 14]. Both I-FABP and L-FABP are elevated in patients with NEC, but also with sepsis, after abdominal surgery or trauma [15–19].

The trefoil factor-3 (TFF-3) is expressed in intestinal tract and is associated with maintaining of mucosal barrier integrity, promotion of mucosal barrier restitution, gastrointestinal inflammation, and cell migration [20–23]. TFF-3 is elevated in patients with severe sepsis and inflammatory bowel diseases. TFF-3 levels correlate with the activity of ulcerative colitis localized to the colonic mucosa, suggesting the site-specific upregulation of TFF-3 in inflamed colonic mucosa [24–26]. Moreover, it was shown that TFF-3 was associated with protective effect on intestinal tract injury in animal model of NEC via protection of excessive apoptosis by the increase of Bcl-2 and reduction of caspase-3 and Bax expression [27].

Serum amyloid A (SAA) is an acute phase inflammatory protein. It increases in innate defence mechanisms in response to infection, inflammation, and trauma. The SAA was suggested to be more sensitive and specific marker of inflammation than C-reactive protein in various inflammatory conditions [28–30]. The SAA has been shown to be useful in diagnosis of various acute diseases, including neonatal sepsis and NEC. Moreover, it can determine the severity of the disease and response to therapy [31–33].

The aim of this study was to investigate the diagnostic potential of these gut-associated and inflammatory biomarkers in the early diagnostics of NEC, their association with clinically relevant and well-established disease-related parameters, and their capacity to predict the disease course. Thanks to their small size, all these biomarkers can pass through the kidney to urine, which gives unique opportunity to constant monitoring and noninvasive measurement.

## 2. Materials and Methods

### 2.1. Patients

In the study, we enrolled 29 patients with suspected NEC and 8 healthy infants without gut surgery and intestinal mucosa disruption as controls. All of them were recruited from the individuals admitted to the Department of Pediatric Surgery of Motol University Hospital, Prague, Czech Republic, between April 2012 and December 2014 (Table 1). The inclusion criteria were stage IA of NEC according to the modified Bell's staging criteria, which are characterized by temperature instability, lethargy, increased gastric residuals, abdominal distension, and occult blood in stool. The patients with suspected NEC were later divided into NEC group (*n* = 20) and sepsis group (*n* = 9) using the standard criteria for NEC (*pneumatosis intestinalis* on X-ray or presence of the gas in the portal vein) or sepsis (suggestive clinical signs, laboratory examination and positive blood culture) [34, 35]. Perinatal asphyxia was evaluated using the guidelines of the American Academy of Pediatrics (AAP) and American College of Obstetrics and Gynecology (ACOG) criteria as described previously [36]. Moreover, in infants who underwent surgery for congenital intestinal malformation (e.g., gastroschisis, volvulus, intestinal or anorectal atresia, and Hirschsprung's disease), urine was collected in 6 h intervals for 48 h after surgery and the levels of the selected biomarkers were compared between these who developed NEC and sepsis. There were no significant differences among groups, except for birth asphyxia in infants suffering from NEC compared with control infants. To analyze the capacity of these noninvasive biomarkers in the prediction of clinical outcome and recovery, we used clinical data from patients' health records and chose length of hospitalization, length of antibiotic (ATB) therapy, and time to full enteral feeding. The median number of the days was a cut off. Therefore, we identified “short” length of hospitalization as less than 19 days, “short” length of antibiotic therapy as less than 10 days, and “short” time to full enteral feeding as less than 9.5 days. The study was approved by the Ethics Committee of the Motol University Hospital, and written informed consent was obtained from parents of all infants included in this study.

### 2.2. Sample Collection and Processing

Urine samples were collected for two days in 6-hours intervals starting at the time of enrolment to the study or already after the surgery for congenital intestinal malformation to evaluate the biomarker dynamics in infants before the suspicion of NEC. These samples were analyzed retrospectively in those who developed NEC or sepsis. The urine was collected either using urine bag connected to an indwelling catheter or from a cotton wool swab placed in the diaper and squeezed through a syringe barrel into a collection tube. The urinary creatinine was measured in each sample immediately after sampling and samples for biomarker analyses were frozen at −20°C.

### 2.3. Enzyme-Linked Immunosorbent Assay (ELISA)

The concentrations of biomarkers were measured by ELISA (Table 2), which is certified for urine analysis by manufacturer. The assays were performed according to the manufacturer's instruction. To eliminate fluctuation in urine excretion, biomarkers were normalized to urinary creatinine and presented as pg/nmol of creatinine.

### 2.4. Statistical Analyses

The differences between studied groups were analyzed by nonparametric Mann-Whitney test. Continuous variables are presented as mean ± standard deviations (SD) and dichotomous variables as percentages. Statistical analyses were performed using GraphPad Prism statistical software (version 8.1.1, GraphPad Software, San Diego, CA, USA) and differences were considered statistically significant at *p* < 0.05. Regression analysis was performed in R and the effect of each biomarker on Akaike information criterion (AIC) was determined in the nnet package (ver. 7.3-12). Next, we performed both backward elimination and forward selection based on AIC to determine the best regression model to discriminate between the two states (e.g., disease type, stage, or outcome). Thus, we found minimum models with best prediction and performance capacity, which were used for construction of receiving operating characteristic (ROC) curves. The ROC curves and their area under curve (AUC) were calculated using ROCR package (ver. 1.0-7). Hierarchical clustering and heat map construction was performed using the Kendall distance calculating method in the ComplexHeatmap (ver 1.3) package for R [37].

## 3. Results

**Figure 1:**
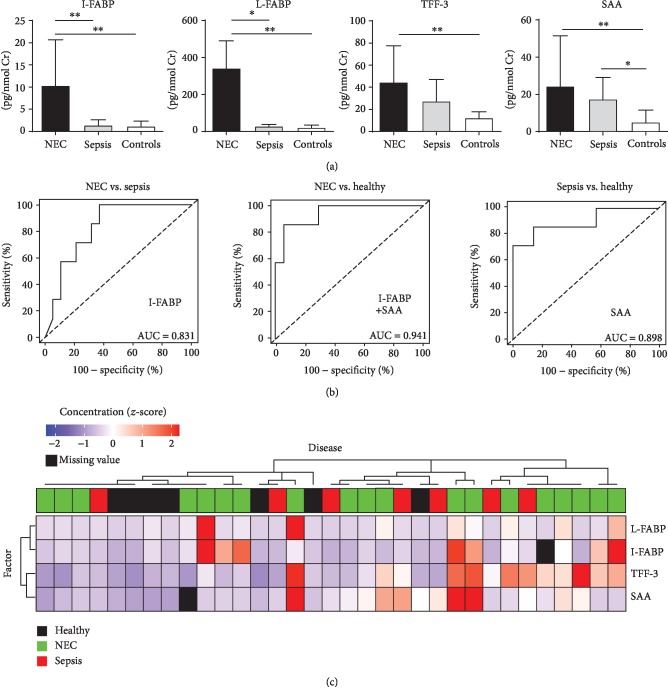
The analysis of I-FABP, L-FABP, TFF-3, and SAA in urine. (a) The comparison of I-FABP, L-FABP, TFF-3, and SAA for patients who will later develop NEC/sepsis or healthy infants in the first 6 hours from the enrolment (^∗^*p* < 0.05, ^∗∗^*p* < 0.01; Mann-Whitney test). (b) Composite ROC curve analysis of the best model found by regression analysis. (c) Heat map and cluster analysis.

During the first 6 hours after the enrolment, infants who will later develop NEC (NEC group) had significantly higher urinary I-FABP, L-FABP, TFF-3, and SAA when compared with healthy infants and higher levels of I-FABP and L-FABP than those who will later develop sepsis (sepsis group) (Figure 1(a)). Similarly as in NEC, SAA was significantly higher in urine of patients with sepsis than that in healthy controls. Urinary I-FABP discriminated future NEC and sepsis, the SAA discriminated sepsis from healthy controls, and the combination of both discriminated NEC from healthy controls (Figure 1(b)). These effects are quite strong despite the fact that the cluster analysis of all these biomarkers did not find particularly strong clustering according to the presence, absence, or type of diagnosis (Figure 1(c)). There were no significant differences in these biomarkers between spontaneous and surgery-related NEC at the time of enrolment (data not shown). Patients who will develop NEC after the surgery for congenital intestinal malformation had higher levels of urinary I-FABP and L-FABP than those who will develop sepsis already in the first 6 hours after the surgery. Moreover, there was steep increase in urinary I-FABP and L-FABP around the time of NEC suspicion in infants who will later develop NEC (Supplementary [Supplementary-material supplementary-material-1]).

**Figure 2:**
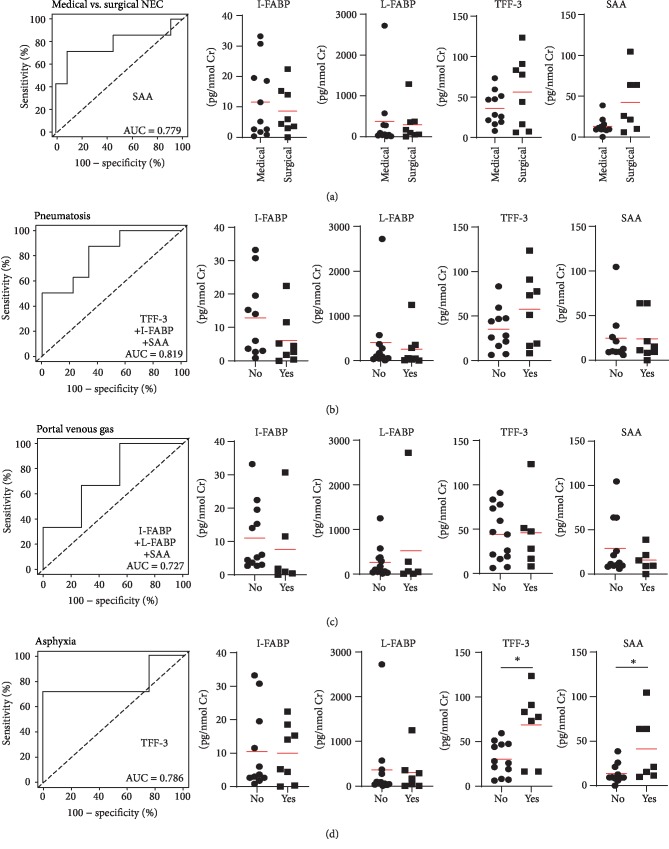
Biomarker patterns discriminating clinical stages. Composite ROC curve analysis of the best model found by regression analysis and quantitative plots of analyzed biomarkers (^∗^*p* < 0.05; Mann-Whitney test).

Next, we analyzed the association of these biomarkers in the first 6 hours after the enrolment with clinically relevant and well-established disease-related parameters. We found that infants who will later develop stage IIIB NEC (“surgical”), which is associated with gut perforation, had elevated both SAA and TFF-3 in comparison with infants with stages IIA, IIB, and IIIA NEC (“medical”) (Figure 2(a)). The SAA was capable to discriminate between medical and surgical NEC. The combinations of TFF-3 with I-FABP and SAA predicted well *pneumatosis intestinalis* and the combination of I-FABP with L-FABP and SAA predicted portal venous gas (Figures 2(b) and 2(c)). Perinatal asphyxia in NEC patients was associated with significantly higher levels of urinary SAA and TFF-3 and the TFF-3 alone was the best discriminating factor (Figure 2(d)).

**Figure 3:**
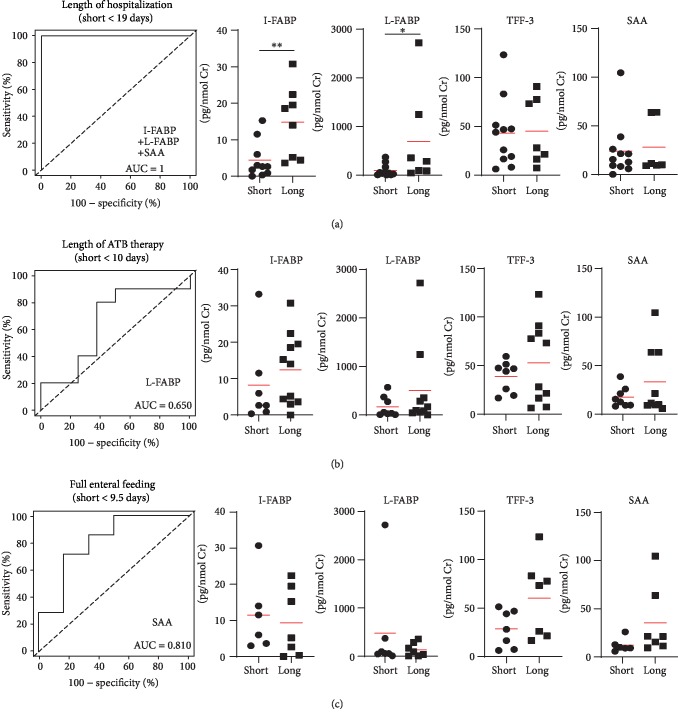
Biomarkers predicting clinical outcome in patients with NEC. Composite ROC curve analysis of the best model found by regression analysis and quantitative plots of analyzed biomarkers (^∗^*p* < 0.05, ^∗∗^*p* < 0.01; Mann-Whitney test).

Next, we analyzed the ability of these noninvasive biomarkers to predict clinical outcome in infants who will develop NEC. The urinary I-FABP and L-FABP were significantly higher in infants with long hospitalization (more than 19 days), and when combined with SAA, they can predict long hospitalization (Figure 3(a)). While L-FABP was associated with the long antibiotic (ATB) therapy in NEC infants, its ability to discriminate between short and long ATB therapy was rather low (AUC = 0.650) (Figure 3(b)). The urinary SAA was the best factor for prediction of the early full enteral feeding (more than 9.5 days) (Figure 3(c)).

## 4. Discussion

In our previous study, we found that both, serum and urinary I-FABP, can distinguish NEC from sepsis in the early stage of the disease, and the addition of I-FABP analysis to the diagnostic gold standard for NEC (X-ray and ultrasound) can significantly increase the sensitivity and negative predictive value [12]. Here, we combine urinary I-FABP with other gut-associated and inflammatory biomarkers with the aim to further improve the differential diagnosis of NEC and to get better insight into the NEC pathophysiology. While several previous studies analyzed these biomarkers in NEC patients, this is the first study analyzing the combination of all these biomarkers not only in early NEC diagnosis but also in association with clinically relevant and well-established disease-related parameters. Moreover, we tested their capacity to predict the disease course and outcome in a noninvasive way. The noninvasive means of urine collection is favourable because it minimizes the stress of the neonate from the repeated blood sampling [38].

In this study, we found that not only I-FABP but also L-FABP, SAA, and TFF-3 were significantly higher in the urine of infants who will later develop NEC in comparison with healthy infants already in the first 6 hours after the NEC suspicion. Their high levels suggest that gut mucosa damage and strong inflammatory response are detectable even before the full spectrum of NEC symptoms is apparent. The increase of I-FABP and L-FABP in NEC infants suggests that they are both associated with the gut epithelium damage. This is in agreement with findings that L-FABP has similar pattern of tissue distribution with higher tissue content than I-FABP [13]. Therefore, L-FABP may be a more sensitive biomarker for detection of gut epithelium damage in early stage of NEC when it is released from the damaged enterocytes, in spite of its association with hepatocellular injury. Our results are thus in agreement with the previous findings, where both analytes were increased in plasma of NEC patients [14]. Among studied biomarkers, the urinary I-FABP was the strongest factor for distinguishing patients who will later develop NEC from those who will develop sepsis. This is in agreement with our previous results and further supports the importance of I-FABP in differential diagnostics of NEC [12]. Since even uncomplicated abdominal surgery could lead to the increase of I-FABP levels [17, 39, 40], we compared all tested biomarkers in patients with surgery-related NEC or sepsis. We found that infants who will develop NEC after the surgery for congenital intestinal malformation had higher levels of urinary I-FABP and L-FABP already in the first 6 hours after the surgery than those who will develop sepsis. This was followed by a substantial decrease after 12 hours from the surgery. At the time of NEC suspicion, there was rapid increase in the levels of urinary I-FABP and L-FABP but only in patients who will later develop NEC. The levels of studied biomarkers in this study did not significantly differ between spontaneous and surgery-related NEC, and the levels of urinary I-FABP and L-FABP between sepsis and control group were comparable.

Our finding that high levels of urinary TFF-3 are associated with NEC is in agreement with previous findings, where the elevated plasma TFF-3 was associated with intestinal damage in NEC [41]. Increased TFF-3 in NEC may represent protective feedback mechanism triggered by the gut injury, which in turn promotes the mucosal barrier restitution [22, 27]. The high serum TFF-3 was described also in the association with the severity, multiple organ dysfunctions, and prognosis of sepsis patients [25, 42]. Thus, we can speculate that increased TFF-3 in sepsis could be a secondary phenomenon caused by the blood redistribution leading to the gut barrier dysfunction, which in turn can contribute to the development of uncontrolled systemic inflammatory response syndrome. We found that urinary SAA is increased in both NEC and sepsis, indicating the central role of the inflammatory response in both diseases. This is in agreement with other studies describing its increase in plasma during both NEC and sepsis [28–30]. Taken together, these results further stress the importance of harnessing our understanding of the biological processes for diagnostic purposes.

Next, we analyzed if these biomarkers are associated with clinically relevant and well-established disease-related parameters in NEC. We found that SAA not only distinguished infants who will develop NEC stage II and III, it can also distinguish infants who will develop the most severe stage associated with gut perforation—stage IIIB NEC (“surgical”) from stage IIA, IIB, and IIIA NEC (“medical”) at the time of NEC suspicion. This is in agreement with a previous study finding urinary SAA correlated with diseases severity [43]. We also found that patient with surgical NEC had higher levels of urinary TFF-3 than those with medical NEC, suggesting the association of TFF-3 levels with more severe intestinal damage in the case of surgical NEC. The combination of gut-associated biomarkers with urinary SAA was the strongest predictor of *pneumatosis intestinalis* (TFF-3, I-FABP, and SAA) and portal venous gas (I-FABP, L-FABP, and SAA) already in the first 6 hours after the enrolment. In our previous study, we showed that currently used gold-standard methods for NEC diagnosis have low sensitivity and negative predictive value. This may cause the late diagnosis of NEC, which is accompanied with poor outcome or risk of death [44–46]. Moreover, infants with suspected NEC are subjected to harmful effects of ionizing radiation by numerous abdominal X-rays [47]. Our new results provide further insight into the early stages of NEC pathophysiology and the association of these biomarkers with clinically relevant and well-established disease-related parameters. Moreover, identification of patients who will develop severe NEC already at the moment of NEC suspicion is of great value for frontline neonatologist and surgeon in monitoring and management of NEC, especially in patients who may benefit from early surgical intervention.

The hypoxic ischemia was described as a one of the risk factor in NEC pathogenesis [8]. It may lead to the acute phase inflammatory response in neonate, development of neuronal damage, and tissue necrosis with devastating clinical outcome [48–50]. We found that among infants who developed NEC, these who were exposed to perinatal asphyxia have significantly higher levels of urinary TFF-3 and SAA than those who were not. Our results are thus in agreement with previous studies considering the SAA as a possible marker for ischemia-related inflammation closely associated with ischemic injuries [49, 51]. The high levels of TFF-3 were previously described in animal model of perinatal asphyxia, where the elevated TFF-3 was considered as the reaction to the tissue injury repair [52].

If these biomarkers reflect the pathogenesis and disease severity, they may predict outcome of the disease. Indeed, we found that higher levels of I-FABP, L-FABP, and SAA predict shorter hospitalization already in the first 6 hours from NEC suspicion. This is in agreement with previous observation that serial I-FABP measurement can predict development of complicated disease and that SAA in serum is useful tool for determining the disease severity and response to therapy in infants with NEC [32, 53]. We found similar trend in length of ATB therapy (L-FABP) and late achievement of full enteral feeding (SAA), both being signs of more severe disease. However, the number of individuals in this study was probably too low to find subtle differences in these parameters and bigger cohorts may be needed. The morbidity and long-term health outcomes among NEC survivors are highly influenced by the pathological stage of NEC and the extent of intestinal damage. The cost associated with both medically and surgically treated NEC is substantially higher than that of matched controls and poses high economic burden for the healthcare [46]. Moreover, there are long-term effects in NEC survivors (e.g., the short bowel syndrome, prolonged administration of parenteral nutrition, and growth and neurodevelopmental impairment) that require further management [54–56]. Our results showed that the gut mucosa damage and inflammatory biomarkers and their combination could be helpful not only in the diagnosis of infants who will develop NEC but also in the prediction of the disease course and outcome and thus can also have treatment and policy implications in management of NEC.

## 5. Conclusion

Development of NEC, its severity, and its consequences can be predicted using the panel of pathogenesis-relevant biomarkers before the symptoms became clinically apparent. The early diagnosis and prediction are useful for the management of NEC by the frontline clinicians.

## Figures and Tables

**Table 1:** Patient's demographics. The data are expressed as number of cases (%) or mean ± standard deviation.

	NEC	Sepsis	Control
Number of infants	20	9	8
Spontaneous NEC	11 (55.0%)	—	—
Surgery-related NEC	9 (45.0%)	—	—
NEC stage II	11 (55.0%)	—	—
NEC stage III	9 (45.0%)	—	—
Sex, male	15 (75.0%)	4 (44.4%)	6 (75%)
Gestational age (weeks)	35.9 ± 3.4	36.7 ± 2.6	38.0 ± 2.8
Birth weight (kg)	2.4 ± 0.9	2.7 ± 0.8	3.2 ± 0.9
Delivery by cesarean section	12 (60.0%)	3 (33.3%)	3 (37.5%)
Birth asphyxia	8 (40.0%)	3 (33.3%)	0 (0.0%)
Congenital heart disease	4 (20.0%)	0 (0.0%)	0 (0.0%)
Age at diagnosis (days)	20.6 ± 33.0	10.1 ± 7.5	11.9 ± 12.6

**Table 2:** List of quantified biomarkers.

Biomarker	Abbreviation	Manufacturer	Cat. no
Intestinal fatty acid-binding protein	I-FABP	Hycult®Biotech	HK406
Liver fatty acid-binding protein	L-FABP	Hycult®Biotech	HK404
Trefoil factor-3	TFF-3	BioVendor	RD191160200R
Serum amyloid A	SAA	Hycult®Biotech	HK333

## Data Availability

The data used to support the findings of this study are available from the corresponding author upon request.

## Abbreviations

NEC: Necrotizing enterocolitis
I-FABP: Intestinal fatty acid-binding protein
L-FABP: Liver fatty acid-binding protein
SAA: Serum amyloid A
TFF-3: Trefoil factor-3
ROC: Receiver operating characteristic
AUC: Area under curve
ELISA: Enzyme-linked immunosorbent assay
GIT: Gastrointestinal tract.

## Supplementary Materials

Supplementary Materials Supplementary figure 1: biomarker dynamics in surgery-related NEC or sepsis. (A) Dynamics of biomarkers 48 hours after abdominal surgery and 48 hours after NEC suspicion. (B) Biomarkers level in the first 6 hours after the surgery for congenital intestinal malformation (^∗∗^*p* < 0.01; Mann-Whitney test).
