# Supplementary figures and images for: Urinary I-FABP, L-FABP, TFF-3, and SAA Can Diagnose and Predict the Disease Course in Necrotizing Enterocolitis at the Early Stage of Disease
Source: J Immunol Res. 2020 Mar 3;2020:3074313. doi: 10.1155/2020/3074313 (PMC7072107; doi:10.1155/2020/3074313)

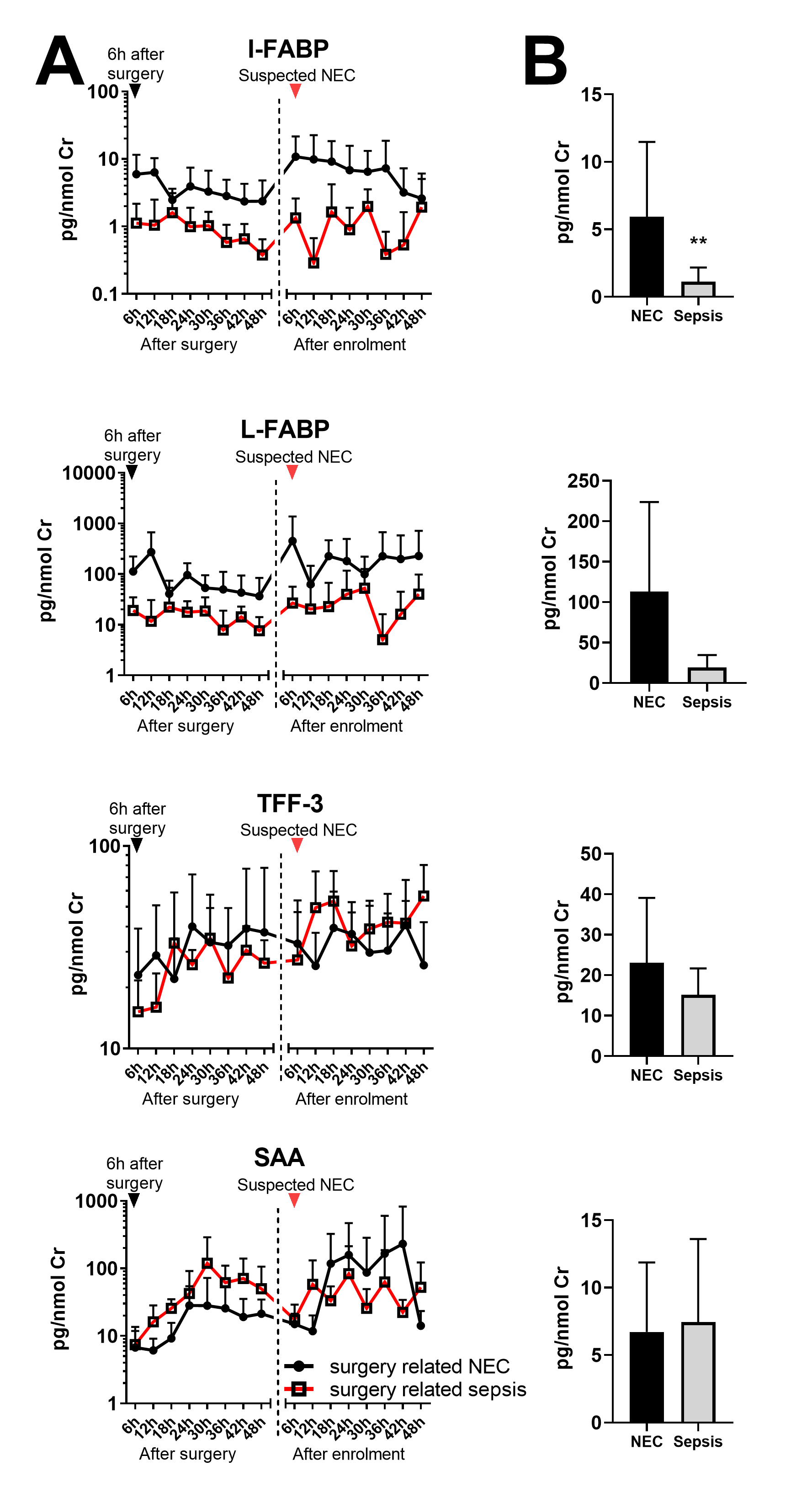

Supplement: Supplementary Materials — Supplementary figure 1: biomarker dynamics in surgery-related NEC or sepsis. (A) Dynamics of biomarkers 48 hours after abdominal surgery and 48 hours after NEC suspicion. (B) Biomarkers level in the first 6 hours after the surgery for congenital intestinal malformation (∗∗p < 0.01; Mann-Whitney test). [file 3074313.f1.png]
